# Harnessing Unconventional T Cells and Innate Lymphoid Cells to Prevent and Treat Hematological Malignancies: Prospects for New Immunotherapy

**DOI:** 10.3390/biom12060754

**Published:** 2022-05-27

**Authors:** Alessandro Allegra, Marco Casciaro, Elena Lo Presti, Caterina Musolino, Sebastiano Gangemi

**Affiliations:** 1 Department of Human Pathology in Adulthood and Childhood “Gaetano Barresi”, Division of Hematology, University of Messina, 98125 Messina, Italy; aallegra@unime.it (A.A.); caterina.musolino@unime.it (C.M.); 2 Department of Clinical and Experimental Medicine, School and Operative Unit of Allergy and Clinical Immunology, University of Messina, 98125 Messina, Italy; gangemis@unime.it; 3 National Research Council (CNR)—Institute for Biomedical Research and Innovation (IRIB), 90146 Palermo, Italy; elena.lopresti@cnr.it

**Keywords:** unconventional T cells, natural killer T cells, innate lymphoid cells, gamma delta T cells, MAIT cells, leukemia, lymphoma, multiple myeloma, vaccination, immunotherapy

## Abstract

Unconventional T cells and innate lymphoid cells (ILCs) make up a heterogeneous set of cells that characteristically show prompt responses toward specific antigens. Unconventional T cells recognize non-peptide antigens, which are bound and presented by diverse non-polymorphic antigen-presenting molecules and comprise γδ T cells, MR1-restricted mucosal-associated invariant T cells (MAITs), and natural killer T cells (NKTs). On the other hand, ILCs lack antigen-specific receptors and act as the innate counterpart to the T lymphocytes found in the adaptive immune response. The alteration of unconventional T cells and ILCs in frequency and functionality is correlated with the onset of several autoimmune diseases, allergy, inflammation, and tumor. However, depending on the physio-pathological framework, unconventional T cells may exhibit either protective or pathogenic activity in a range of neoplastic diseases. Nonetheless, experimental models and clinical studies have displayed that some unconventional T cells are potential therapeutic targets, as well as prognostic and diagnostic markers. In fact, cell-mediated immune response in tumors has become the focus in immunotherapy against neoplastic disease. This review concentrates on the present knowledge concerning the function of unconventional T cell sets in the antitumor immune response in hematological malignancies, such as acute and chronic leukemia, multiple myeloma, and lymphoproliferative disorders. Moreover, we discuss the possibility that modulating the activity of unconventional T cells could be useful in the treatment of hematological neoplasms, in the prevention of specific conditions (such as graft versus host disease), and in the formulation of an effective anticancer vaccine therapy. The exact knowledge of the role of these cells could represent the prerequisite for the creation of a new form of immunotherapy for hematological neoplasms.

## 1. Introduction

### 1.1. General Aspects on Unconventional T Cells

In recent years, more attention has been paid to the unconventional T cell subsets and their role in anti-tumor immunity, especially in hematology malignancies, due to new findings on the topic. Unconventional T cells, namely, γδ T cells, MAIT cells, and iNKT cells (invariant natural killer T) as a part of NKT cells, have features of both innate and adaptive immunity that can be summarized in three main points:

- rapid responses to innate immune cells due to antigen-independent activation thanks to cytokines and ligand recognition;
- non-classical MHC to innate immune cells;
- the exhibition of limited T cell antigen receptor (TCR) repertoires, recognizing unconventional peptide antigens as a distinctive feature.

Depending on the expression of the delta chain variable region segment of their TCR, γδ T cells are distinctive in subpopulations prevalently found in tissue or in blood. Vδ1-positive cells are the most abundant population in adult peripheral tissues, including the gut, skin, and liver. Vδ2-positive cells are found mainly in blood, accounting for about 0.5–5% of total CD3+ cells and are usually paired with a Vγ9 chain. Finally, as the non-Vδ2 population, there are Vδ3-positive cells accounting for only 0.2% of peripheral CD3+ cells, but their frequency can increase in lupus patients or in cytomegalovirus (CMV) and HIV-infected patients. Over the last few years, alongside Vδ2+ T cells, the subpopulation of Vγ9-negative T cells clonally expands upon CMV infection and shows effector function [1].

MAIT cells are abundant in humans representing up to 10% of circulating CD3+ T cells in the peripheral blood, but they are preferentially localized in tissues and mucosa, representing up to 45% of liver T cells [2]. A minor population representing only 0.1–1% of human T cells in the blood and liver are natural killer T (NKT) cells. They are divided into two subtypes: type I and type II NKT cells. Type I NKT cells are more commonly referred to as invariant NKT (iNKT) cells because their TCR is composed of an invariant α-chain (V-α24/J-α18 in humans) bound to a limited array of β-chains. To date, they represent the more studied subtype amongst NKT as the knowledge of type II NKT cells is currently limited given the inaccurate and imprecise methods used to detect them.

Different from αβ T cells and unconventional T cells, there are the innate lymphoid cells (ILCs) that develop from common lymphoid progenitor cells (CLPs) but have no rearranged antigen-specific receptors and, in contrast, are enriched on their surface of CD127 (IL-7Rα) [3]. Classically, they can be divided into three groups on the basis of the expression of transcriptional factors and cytokines responding differently to several stimulations [4].

So, we distinguish group 1 ILC (ILC1) with Th1-like properties which express transcriptional factor T-bet and respond to IL-12, IL-15, and IL-18 stimulation. Group 2 ILC (ILC2) can generate Th2-like cytokines under IL-25 and thymic stromal lymphopoietin (TSLP) induction and are characterized by transcriptional factors RoRα and GATA3. Group 3 ILC (ILC3) is defined by the expression of RORγt and AHR-producing IL-22 and/or IL-17 under the stimulation of IL-23 and/or IL-1β. ILC3 can be divided into two subgroups based on their expression of the natural cytotoxicity receptor (NRC) NKp44; indeed, NKp44+ ILC3 is the main producer of IL-22 [5]. Finally, a novel recognized subset is regulatory ILC (ILCreg) which secretes IL10 and TGF-β (acting in an autocrine manner too) under the stimulation of IL-2 in the presence of transcriptional factors Id3 and SOX4 [6].

### 1.2. Activation and Functions of Unconventional T Cells

Human γδ T cells display broad reactivity to many types of viruses and bacteria, showing potent cytotoxic functions and cytolytic activity via the expression of perforin, granzymes, and natural cytotoxicity receptors. γδ T cells play a role to defend against bacteria [3] and CMV infection and have been postulated to play an in vitro protective role against coronavirus infection [7]. To date, the blood ratio of immature neutrophils to γδ T cells has been considered as a good prognostic marker for severe COVID-19 [8,9].

Similarly, MAIT has also been shown to have a protective role in immunity against human virus infection, such as hepatitis C virus in which in vitro activated MAIT cells were shown to suppress virus replication [10]. Even though the links with clinical outcome are unclear, MAIT shows signs of activation during SARS-CoV-2 infection, as reported in many pieces of research [11]. In a similar fashion, NKT cells are involved in microbial infection, demonstrating anti-viral immunity against HIV or COVID-19 through direct cytotoxicity, for instance [12].

In different manners and at different levels, all unconventional T cells can contribute to tumor surveillance because they populate tissues and mucosa, and also circulate in the blood. The expression of multiple receptors and the fact that they can directly respond to cytokines in a TCR-independent manner demonstrates their primary role in defense against tumor development. For example, innate receptors, namely NKG2D, NKp30, and NKp46 exhibited on Vδ1+ T cells, have been studied in detail given their relevance, as demonstrated in primary multiple myeloma patients [13].

However, through their TCR, they recognize “unconventional antigens” presented by MHC-like molecules, such as BTN, MR1, and CD1, with an immediate response.

In particular, phosphoantigens activate Vγ9Vδ2 cells by binding butyrophilin 3A1 (BTN3A1) [14], which is ubiquitously expressed on tumor cells and cooperates with BTN2A1 to date, and is considered fundamental for the activation of γδ T cells [15]. Differently, Vδ1 cells recognize various stress-related ligands, including MICA, MICB, ULBP, and CD1c and CD1d glycoproteins [16], through MHC class I, establishing their reactivity towards phycoerythrin [17], and more recently, ephrin type-A receptor 2 [18], a non-MHC-like protein, similar to annexin A2 and endothelial coupled protein-C receptor, recognized by Vδ3 and Vδ5, respectively [19,20]. MAIT shows the capacity to respond to antigens derived from microbial vitamin B biosynthesis intermediates, such as activating vitamin B2 (riboflavin) metabolites, presented by MHC class-I-related molecule MR1 without requiring the presence of b2-microglobulin [21]. In opposite to this activation mechanism, MAIT cell activation can be performed in a TCR-independent manner by responding to IL-12 and IL-18 directly. In the same fashion, type I NKT cells recognize glycolipid antigens (most notably alpha-galactosylceramide (α-GalCer)) presented by another class-I-like molecule, CD1d, while type-II NKT cells recognize mammalian glycolipid sulfatide, which is produced at high concentrations in neuroendocrine tissue [22].

ILCs can maintain tissue homeostasis and inflammation by releasing cytokines. Indeed, they show protective responses against microorganisms in lymphoid tissue formation and in tissue remodeling after damage. For example, consistent studies demonstrated their involvement in inflammatory bowel disease (IBD), similar to γδ T cells, with a predominant frequency of ILC1 and ILC2 in patients with CD and with UC, respectively [23,24].

Particular relevance is attributed to the ability of ILC and unconventional T cells to produce IL17, which is considered a crucial cytokine due to its levels being elevated in various inflammatory conditions, including sepsis, pneumonia, systemic lupus erythematosus, rheumatoid arthritis, allograft rejection, and cancer.

Indeed, there is a subset of MAIT that can express CD161^hi^ that is associated with the production of IL17, which has various implications for antiviral immunity [25]. Tumor-infiltrating IL17-producing γδ T cells are associated with worse prognosis in patients with a solid tumor; thus, there is a need for more in-depth studies [26]. IL-17-producing NCR-negative ILC3 can increase frequency in the gut of IBD patients and is involved in tumor development given the association of neutrophil recruitment that can disrupt junction proteins, such as E-cadherin and JAML, leading to epithelial permeability, and either increasing the inflammation state or promoting angiogenesis [27].

The purpose of this review was to evaluate the presence of quantitative and functional alterations of unconventional T cells in the various hematological malignancies and to present the data existing in the literature on the possibility of modulating cell activity for the prevention or treatment of liquid tumors.

### 1.3. Search Strategy

A literature search was conducted for English language publications in MEDLINE (via PubMed). A time restriction was not applied to the search strategy. The search terms were “unconventional T cell”, “innate lymphoid cell”, “MAIT cell”, “iNKT cell”, “natural killer cell”, “γδ cell” AND “acute myeloid leukemia”, “acute lymphoblastic leukemia”, “chronic myeloid leukemia”, “multiple myeloma”, “chronic lymphocytic leukemia”, and “lymphoma”. Studies were accepted from any country. The population of interest was pediatric and adult patients, given the diverse incidence of different hematological neoplasms at different ages.

## 2. Acute Leukemia

Acute leukemia is a hematological malignancy which influences differentiation, proliferation, and cell cycle progression in myeloid or lymphoid precursors. Although chemotherapy has been the traditional form of treatment for these diseases, this approach augments infection vulnerability, comorbidities, and immunosuppression. Moreover, immunotherapies demonstrate hopeful treatment opportunities for leukemia [28].

**Figure 1:**
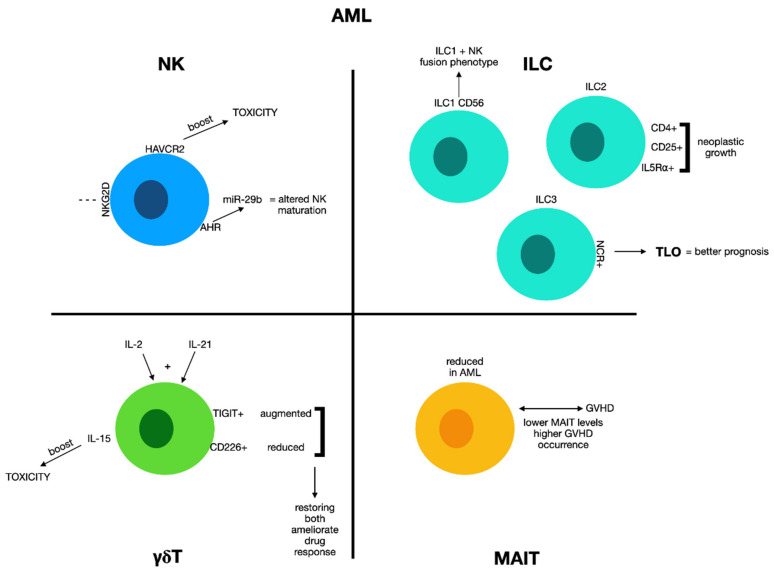
Acute myeloid leukemia (AML) unconventional immune cell scenario. NK: The NK receptor NKG2D can make tumor cells vulnerable to NK cell-mediated lysis. The systemic reduction of NKG2D on the NK surface of tumor subjects is reported, causing an alteration in NKG2D-mediated NK cell function. Hepatitis A virus cellular receptor 2 (HAVCR2 or TIM-3) is intensely present on NK cells in AML subjects, associated with augmented cytotoxic activity and with a better prognosis. Moreover, AML blasts can stimulate the aryl hydrocarbon receptor (AHR) system that augments miRNA-29b production in NK cell precursors, altering their maturation process and activity. ILC: The CD56 innate cell set has mixed phenotypic and transcriptional characteristics of traditional ILCs and lytic NK cells. These CD56 ILC1-like cells have a relevant cytotoxic ability. On the other side, the co-transfer of CD4+CD25+IL5Rα+ILCregs stimulates neoplastic growth. Finally, the presence of NCR+ ILC3 in TLO is related to a better prognosis. γδ T: Results indicate an altered presence of TIGIT and CD226 on γδ T cells with an increase in TIGIT+ γδ T cells and a reduction in CD226+ γδ T cells in subjects with de novo AML, but restoring both can ameliorate a drug response. IL-2 and IL-21 stimulate γδ T cells and IL-15 boosts it toxicity. MAIT: A reduction in AML. In addition, lower MAIT levels favor higher GVHD occurrence.

However, excellent knowledge of the state of the immune system, and in particular of the unconventional effectors, seems to be an indispensable prerequisite for the effective treatment of leukemia, as immune dysfunction has been effectively characterized in this condition and has shown to play a role in both disease severity and progression. In subsequent sections, we will evaluate how the different effectors of innate immunity are modified in the various forms of leukemia and how the modulation of their activity can be used to treat these pathologies (Figure 1).

### 2.1. Natural Killer Cells and Acute Myeloid Leukemia

It has been reported that acute myeloid leukemia (AML) subjects present notably smaller amounts of circulating NK cells with respect to normal subjects that show a relationship with a poorer outcome [29].

In addition to decreased quantities of NK cells, Chretien et al. stated that AML subjects often have a less mature circulating NK cell phenotype, and they evaluated CD57 and KIR expression, which had inferior overall survival (OS) compared to AML subjects expressing a more mature NK cell profile both in the bone marrow and peripheral blood [30]. These results are supported by other studies which display a block in NK cell maturation in animal experimental models of AML, evidenced by their incapacity to pass from stage 2 (CD27+CD11b−) to stage 3 (CD27+CD11b+) NK cells in the spleen [31]. Significantly, this blockade was revocable, indicating that treatment might re-establish mature effector NK cells.

Changes in the maturation process are also demonstrated by the presence of an altered operative function. In fact, mature NK cells extracted from the peripheral blood of AML subjects show a low presence of major stimulating NK receptors, an augmented presence of CD94/NKG2A inhibitory receptors, reduced generation of IFN-γ and TNF-α, and diminished cytolytic abilities [32,33].

A modification of NK cell activity could be fundamental in the onset of AML, particularly in its secondary forms. Several experimentations have reported genetic susceptibilities reaching AML with coexistent NK cell alteration. The genetic deficit of the transcription factor GATA2 disposes patients to AML [34,35] and provokes relevant NK cell alterations, especially deficiency in CD56bright cells with the maintenance of the CD56dim compartment [36,37].

The augmented incidence of AML in subjects with altered NK cell activity appeared to be partially correlated to the deficiency of detection and, consequently, the inability to kill tumor cells. Several mechanisms appeared to be capable of justifying such a situation. For instance, ligands of the activating NK receptor NKG2D make tumor cells vulnerable to NK cell-mediated lysis. However, tumor cells utilize different mechanisms to elude NKG2D-mediated surveillance, such as NKG2D ligand shedding, causing a decreased surface expression amount. Furthermore, a systemic reduction in NKG2D on the NK surface of tumor subjects was reported in several experimentations and was ascribed to soluble NKG2DL (sNKG2DL). In fact, in AML subjects, the presence of sNKG2DL is correlated with a reduction in the surface NKG2D expression, causing an alteration in NKG2D-mediated NK cell function [38]. Soluble ligands of N-cell-stimulating receptors can also be found joined to tumor-originated exosomes in leukemic subjects’ serum, provoking the inhibition of NK cell stimulation [39]. Other soluble molecules, such as transforming growth factor-beta1 (TGF-β), may also have remarkable repressive impacts [40], and sera isolated from AML patients have been reported to include micro-vesicles carrying TGF-β at their surface, which can induce a change in NK cell activity.

Co-inhibitory receptors present in T cells significantly affect the institution of a clinically significant reduction in the immune response of several tumors or, in some conditions, the enhancement of the immune activity. Hepatitis A virus cellular receptor 2 (HAVCR2 or TIM-3) is intensely present on NK cells in AML subjects, associated with augmented cytotoxic activity and a better prognosis [41]. AML blasts may stimulate the aryl hydrocarbon receptor (AHR) system that augments miRNA-29b production in NK cell precursors, altering their maturation process and activity [42]. NK cells offer multiple capacities to interfere with tumor development because they have cytotoxic and regulator activity. As an anti-tumoral immune cell, the analysis through the expression of specific cell surface markers, such as TIM3, needs more in-depth prospective studies to assess the its use for AML patients, as well as optimized therapies currently administrated for patients with solid tumor.

In parallel, the prospect of adjusting the operative modifications of the NK cells of AML patients and employing these cells to cure leukemia nowadays seems like an actual therapeutic possibility. NK cell capacity is used to destroy neoplastic cells, i.e., MHC class-I-defective cells, in diverse therapeutic strategies, comprising the administration of lymphokine-activated NK cells, hematopoietic stem cell transplantation (HSCT), or NK cells modified to present chimeric antigen receptors (CAR) for the treatment of high-risk acute leukemia [43,44,45].

In a study performed on AML subjects with refractory disease, Björklund et al. assessed the efficacy of IL-2-stimulated allogeneic NK cells [46]. Their findings sustain the hypothesis that NK cells may exert anti-leukemic activity on AML subjects, even in chemotherapy-resistant subjects, and these data were confirmed by a different study performed on older AML patients [47]. Responding subjects showed less marked stimulation of CD8^+^ T cells and lesser amounts of inflammatory cytokines after NK-cell administration. Furthermore, NK cells transitorily persist and undergo in vivo maturation into antileukemic effector cells.

Different types of NK cell treatments presently being evaluated on AML patients comprise expanding blood NK cells which employ K562 feeder cells and originating NK cells from induced pluripotent stem cells. Encouraging results were attained in a phase 1 trial employing expanded NK cells ex vivo utilizing membrane-bound IL21 expressing K562 feeder cells. The study displayed only a few cases of infusion-correlated reactions, although some subjects presented bland graft versus host disease (GVHD)-related signs [48].

Different strategies in which NK cells may be essential for leukemia treatment have been implemented in the haplo-hemopoietic stem cells transplant (HSCT) setting to treat high-risk leukemia. In this group of patients, NK cells have been reported to have a relevantly significant impact on leukemia cells and the onset of infections [49].

An important role of NK cells has been highlighted in AML transplant therapy [50,51]. This relationship is because an essential graft management in this type of transplant is the massive T lymphocyte reduction to prevent a grave GvHD. In this highly incompatible HSCT, NK cells have a crucial impact on anti-leukemia activity in the absence of T cells. This therapeutic activity is correlated with NK alloreactivity, secondary to a KIR-HLA difference in the donor versus recipient direction. Remarkably, the dimension of the alloreactive NK cells was reported to relate to more effective anti-leukemia activity and the survival possibility. This T-diminished haplo-HSCT also allows the opportunity to evaluate in vivo the NK cell expansion from CD34 + HSC [52]. The first NK cell set demonstrable in the peripheral blood of HSCT-recipient is CD94/NKG2A+, while KIR+ NK cells emerge in the peripheral blood after 6–8 weeks. A new transplantation setting has been implemented [53,54]. This is founded on the selective reduction of TCRγδ + T cells, which are responsible for GVHD and B cells; thus, the administered mononuclear cells, in addition to CD34+ cells, also comprise mature alloreactive NK cells and TCRγδ + T cells, both provided with anti-leukemia function [55,56].

As reported above, a prominent development in leukemia treatment is constituted by the employment of chimeric antigen receptor (CAR)-engineered NK cells which, differently from CAR-T cells, may derive from allogeneic donors since they do not provoke GVHD. Clinical experimentations employing CAR NK cells have displayed only a few cases of treatment-correlated cytokine release syndrome or infusion reactions, collateral effects generally caused by CAR T cell treatment. The first phase 1 research employing CD33-CAR NK-92 cells in relapsed/refractory AML subjects did not find dose-limiting toxicities at up to 5 billion cells per subject [57]. However, a shortcoming of employing this type of cell, i.e., a tumor cell line, is that it is necessary to irradiate the cells to control growth in the recipient, and this procedure causes a short half-life of cells; thus, multiple administrations are required to obtain long-lasting responses [57].

### 2.2. Innate Lymphoid Cells and Acute Myeloid Leukemia

It was reported that the amount of innate lymphoid cells (ILCs) in AML subjects is remarkably diminished after chemotherapy with respect to normal subjects [58], and other studies have confirmed this finding. However, research assessed the amount of total ILCs in the peripheral blood of normal subjects and AML patients. The findings displayed that ILCs were considerably altered at diagnosis in relation to the number, subsets, and activity, while ILC normality was in part recuperated in subjects responsive to treatment, proposing that ILC dysregulation characterizes AML regardless of chemotherapy. Moreover, authors stated a positive relationship between the amount of peripheral blood in ILCs and the rate of circulating leukemic blasts. They found no distinction between their number in the bone marrow and peripheral blood at disease onset. Fascinating are the data on the functionality of these cells that appeared drastically altered in the cytokine production upon in vitro stimulation compared to ILC from normal subjects [59].

A different experimentation recognized a CD56 innate cell set holding mixed phenotypic and transcriptional characteristics of traditional ILCs and lytic NK cells. These CD56 ILC1-like cells have a relevant cytotoxic ability that is reduced in AML subjects at the onset of disease but is re-established after remission. To explain the mechanism of alteration and restoration, it has to be reminded that their killing ability is KIR-independent and is due to the expression of NKp30, NKp80, TRAIL, and NKG2A. The presence of leukemic blasts intensely reduces their cytotoxic activity, likely by decreasing the presence of cytotoxic-correlated molecules. Remarkably, CD56 ILC1-like cells are present in the NK cell preparations employed in NK transfer-based clinical experimentations, and repairing their activities with anti-NKG2A antibodies might present a new approach for enhancing immune treatments [60].

On the other hand, other groups of ILCs such as type 2 innate lymphoid cells (ILC2s) may be altered as far their amount and function in AML subjects. One such study demonstrated that mesenchymal stromal cells (MSCs) from AML subjects or normal MSCs with an increased expression of cyclooxygenase-2 (COX2) stimulated the growth of co-cultured hematopoietic stem and progenitor cells (HSPCs), which can be avoided by use of COX2 knockdown or TM30089, an antagonist of the PGD2 receptor CRTH2. The study demonstrated that the PGD2-CRTH2 pathway operates on ILC2s, stimulating their growth and inducing them to generate IL-5 and IL-13. The interruption of the PGD2-stimulated ILC2reg pathway inhibited growth of HSPCs. On the contrary, co-transfer of CD4 + CD25 + IL5Rα + Tregs stimulated neoplastic HSPC growth and accelerated leukemia expansion in xeno-transplanted animals. Thus, the PGD2-activated ILC2-Treg axis could be an important therapeutic target for AML [61]. Analogous results have been obtained in acute promyelocytic leukemia, a form of AML in which tumor-originated PGD2 and NKp30-BH76 engagement stimulates ILC2s produce IL-13 that induces myeloid-derived suppressor cells (MDSC) [62]. MDSC can inhibit cytotoxic T lymphocytes (CTLs) development in vitro, and to generate of antigen-specific CD8+ T cell tolerance in vivo [63].

The effects of ILC2s have also been recognized in allogeneic transplantation. Steroid-non-responsive acute (a)GVHD is the most important complication for AML subjects experiencing allo-HSCT, with only 15% of these subjects alive after 1 year. Previous studies demonstrated that the administration of donor ILC2 cells could both avoid and cure aGVHD of the gut with no impact on the graft-versus-leukemia response [64]. However, this clinical strategy is unwieldly, as it can necessitate the production of donor-originated ILC2 cells for each patient. So, the possibility to employ third-party ILC2 cells can offer ready-to-use preparation to avoid aGVHD. A different study confirmed that third-party ILC2 cells augment the survival rate of allo-HSCT patients. The protocol provided for four weekly administrations of ILC2 cells and ILC2 cell activities was entirely absent if the cells could not produce IL-13 and amphiregulin. The possibility to produce third-party ILC2 cells guarantees a new and effective possibility to prevent aGVHD [65]. The cellular therapies based on ILC2 constitute an advanced step towards personalized medicine.

A different set of ILCs cells which also seems to be modified in AML subjects is that of ILC3s. Natural cytotoxicity receptor-positive (NCR+) ILC3 cells participate in the development of tertiary lymphoid organs (TLO) at the tumor sites, and it has been reported that the presence of TLO is correlated with better outcomes.

One study demonstrated a reduction in NCR+ ILC3s but not NCR- ILC3s in peripheral blood samples of AML subjects at diagnosis [66].

However, the uncertainty of ILC effects comprising ILC3 in the progression of tumors may present significant uncertainties regarding their possible therapeutic employment in AML, although their ability to stimulate tissue repair and protect against pathogens may be advantageous in AML subjects undergoing chemotherapy or radiotherapy before HSCT. These treatments provoke tissue injuries as critical intestinal mucositis [67]. These harms can worsen with allo-HSCT following the occurrence of GVHD due to donor T lymphocytes [66]. An animal experimental model of aGVHD demonstrated that host-originated IL-22 could avoid the onset of GVHD, and that gut ILC3 cells are the principal makers of IL-22 after total body irradiation therapy [67,68,69]. Furthermore, ILC3-originated IL-22 can also promote thymic epithelial cell recovery, thus permitting a prompter re-formation of the T cell subset [70].

Furthermore, ILCs may participate in the repair of the mucosal health, and also in the restructuring of mucosa-associated TLO as reported above, and in this situation, it has been demonstrated that CD34+ cells, employed as a font of hematopoietic precursors in HSCT, differentiate towards ILC3, and these cells are more present in the lymphoid progenies of CD34+ precursors originated from umbilical cord blood or bone marrow [71,72].

A further set of ILCs seems to be changed in AML subjects, the regulatory innate lymphoid cells (ILCregs). A few years ago, a sub-group of ILC cells with phenotype of Lin-CD45+ CD127+ IL-10+ was detected in the gut and identified as ILCregs [73]. While the aspect of these cells remembers lymphocytes, they do not present CD4 and FoxP3, and so they are not Tregs [74]. ILCregs present ILC antigens (such as CD25 and CD90) and have an increased expression of IL-2R gamma and SCA-1, but they do not present ILC1 markers (such as NK1.1 or NKp46), ILC2 molecules (such as ST2 and KLRG1), or ILC3 markers (such as NKp46, CD4, and RORγt) [75].

A number of ILCreg cells in AML subjects were remarkably reduced with respect to findings in healthy controls. Furthermore, examining miRNAs from ILCregs-correlated genes comprising TGFBR1, TGFBR2, ID2, ID3, IL2RB, IL3RG, and SOX4 displayed 34 miRNA from plasma samples and 14 miRNAs from BM cell samples, which were different in AML subjects and healthy donors [76]. These findings confirmed both the reduction and the functional alterations of ILCregs in AML patients [76].

### 2.3. γδ T Cells and Acute Myeloid Leukemia

A study assessed the composition of γδ T cell subgroups in AML subjects of diverse clinical statuses by evaluating the immune checkpoint co-inhibitor T cell immunoglobulin and immunoreceptor tyrosine-based (TIGIT) inhibitory (motif domain) and its co-stimulatory receptor CD226 [77]. Results stated an altered presence of TIGIT and CD226 on γδ T cells with an augment in TIGIT+ γδ T cells and a reduction in CD226+ γδ T cells in de novo AML subjects, while TIGIT− CD226+ γδ T cells were reinstated in AML subjects who attained a complete response after treatment. Interestingly, subjects who presented with higher amounts of TIGIT+ CD226− γδ T cells showed shorter overall survival for non-acute promyelocytic leukemia, and this could be recognized as a new prognostic marker [77]. In agreement with the results of this study, a recent meta-analysis of gene expression results performed on nearly 18,000 cancer patients comprising hematological tumors and solid tumors recognized infiltrating γδ T cells as the most relevant element correlated with favorable outcomes [78,79]. Transcriptomic studies showed an augment of Vγ9Vδ2 cells and a subset of γδ T cells in the group of leukemia patients [80], and this increase was positively correlated with improved outcomes for CLL and AML patients only.

On the basis of these data, γδ T cells have been taken into consideration as a possible cell source for T cell-mediated antileukemic treatment and augmenting Vδ1 T cells in AML subjects might be useful [81,82]. In fact, adoptive transfer and the in vivo stimulation of γδ T cells are secure therapeutic strategies that can reach relevant clinical responses [83]. A relevant vantage with employing γδ T cells is that they are improbable to provoke GVHD, permitting them to be produced from normal subjects and administered in an allogeneic set of patients as a ready-to-use treatment [84].

However, despite the above, according to some studies, attention should be given to patients undergoing allogeneic transplantation. In an animal model of GVHD, some studies have reported the alloreactive capacity of γδ T cells which can cause GVHD onset; transgenic mice presenting a great amount of γδ heterodimers on peripheral T cells responded to non-classical MHC class lb and provoked aGVHD when employed as donors in allo-HCT animal models [85]. On the contrary, the diminished occurrence of GVHD was observed in animals treated with anti γδ TCR antibodies or in γδ deficient animals. This was justified by decreased donor T cell development and diminished allogeneic stimulatory ability of DCs [86].

Numerous methods have been used to enhance the therapeutic effect of these cells, and a study assessed the effects of IL-15 on γδ T cells and employed a stimulatory tool in the ex vivo growth of γδ T cells for adoptive transfer in AML patients. The addition of IL-15 to γδ T cell cultures induced a more activated phenotype with a more evident Th1 polarization, and an increased cytotoxic capacity [87].

The effect of IL-15 on γδ cells has also been used in different experimentations, such as the attempt to harness γδ T cells by a dendritic cells (DC) vaccine to amplify the anti-tumor efficacy of vaccination [88]. In a study, authors address soluble IL-15 produced by IL-15 DCs as the main factor responsible for the IL-15 DC-mediated γδ T cell stimulation. Therefore, the use of IL-15-producing DC cells could make DC-based cancer vaccines more effective through the participation of γδ T lymphocytes in the anti-leukemic immune response [89].

A novel approach to augment γδ activity implicates the employment of artificial antigen-presenting cells (aAPCs) originated from the K562 cell line [90]. These leukemic feeder cells were altered to present several molecules on their membrane, such as CD19, CD64, CD86, and IL-15. When γδ T cells were co-cultured and IL-2 and IL-21 were added, there was an exceptional polyclonal expansion and most of these cells presented different γδ TCR domains and could destroy leukemic cells via TCR, NKG2D, and DNAM-1 [85,90]. As part of these methods, also for γδ T cells, there was the possibility of targeting CD19 antigen-positive leukemia cells with promising efficacy [91]

### 2.4. MAIT Cells and Acute Myeloid Leukemia

In the context of hematological neoplasms, MAIT cells are, for the most part, unexplored. However, as these cells are extremely abundant and have a great cytolytic capacity, MAIT cells might be excellent agents for immunotherapeutic treatment in leukemic patients.

In a prospective experimentation involving 216 AML subjects, circulating MAIT cells were measured before and after chemotherapy [92], and it was stated that MAIT cells displayed a reduction in AML subjects with respect to normal subjects. Moreover, after induction treatment, leukemic patients showed a further severe decrease in MAIT cell numbers, with improvement after one month. Interestingly, a relationship between reduction in MAIT cells number and unfavorable cytogenetic signature was also found, suggesting a correlation between MAIT cells and AML progression [92].

Even in the case of MAIT cells, the most remarkable experimentations in AML subjects are those correlating to their effects in the group of AML subjects experiencing allogeneic transplantation. Solders et al. reported a reduction in MAIT cell counts in subjects with grade 2–4 GVHD versus subjects with grade 0–1 GVHD [93]. Analogous findings have been reported by other authors such as Bhattacharyya who registered reduced MAIT cell counts on day 30 after allo-HCT in leukemic subjects with grade 3–4 GVHD [94]. MAIT cell reconstitution might be considered as a predictor of GVHD, as lower MAIT cell counts on day 60 after transplant have been related to the onset of aGVHD in a study performed on pediatric and adult subjects receiving bone marrow transplantation [95,96,97].

### 2.5. Acute Lymphoblastic Leukemia

#### 2.5.1. Natural Killer Cells and Acute Lymphoblastic Leukemia

Acute lymphoblastic leukemia (ALL) is a hematologic malignancy with highly aggressive features, which is inclined to relapse, and has a bad outcome and limited clinically efficacious treatments [98].

**Figure 2:**
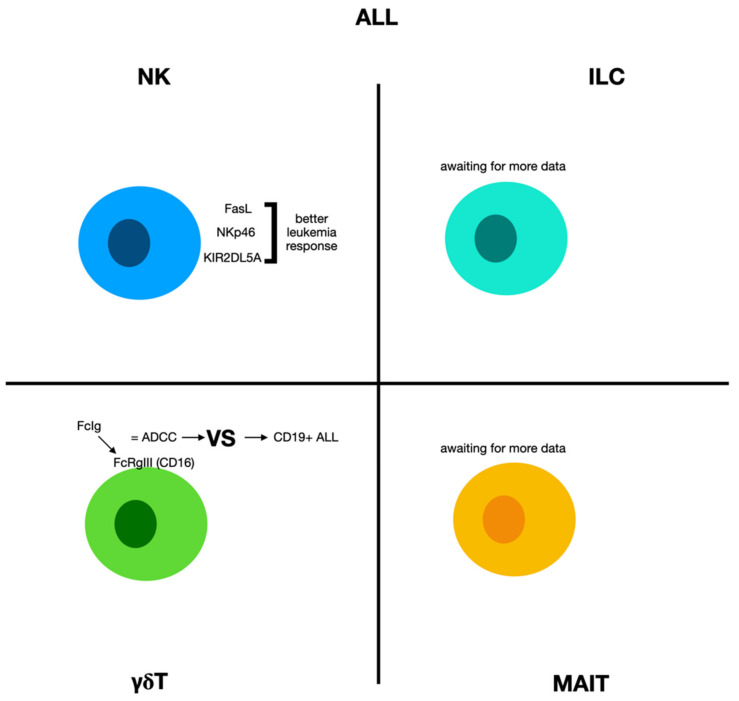
Acute lymphoblastic leukemia (ALL) unconventional immune cell scenario. NK: NK cells presenting FasL, NKp46, and KIR2DL5A in ALL subjects was related to an enhanced leukemia response. γδT: Fc receptor FcRgIII (CD16) joins to the Fc portion of immunoglobulins and provokes anti-leukemic actions through antibody-dependent cell cytotoxicity (ADCC) effects, similar to NK cells.

However, only a few studies have evaluated the function and effects of unconventional T cells in ALL progression (Figure 2). In any case, the number of NK cells in the bone marrow of ALL at the onset was associated with enhanced response to treatments and augmented leukemia remission percentages [99]. Furthermore, the prevalence of stimulated NK cells presenting FasL, NKp46, and KIR2DL5A in ALL subjects was related to enhanced leukemia responses after the administration of cytarabine, methotrexate, and hydrocortisone [100]. Thus, many studies characterizing NK in ALL patients are required to find new targets for combined immunotherapies.

#### 2.5.2. γδ Cells and Acute Lymphoblastic Leukemia

The main mechanism for anti-leukemic effect of γδ T cells is the presence of Fc receptor FcRgIII (CD16). In fact, CD16 can stimulate Vg9Vd2 T cells provoking TNF-a production, which in turn is regulated by CD94 NK receptors. This molecule joins to the Fc portion of immunoglobulins and provokes anti-leukemic actions through an antibody-dependent cell cytotoxicity (ADCC) effect, similar to NK cells [101]. The effectiveness of the γδ T cell-originated ADCC against CD19+ ALL was confirmed by employing a CD19 antibody [102], as well as a so-called “triplebody” with two binding sites for CD19 and one for CD16 [103], and a combination of others combination can permit the rapid lysis of leukemic blasts.

### 2.6. Chronic Myeloid Leukemia

#### 2.6.1. Natural Killer Cells and Chronic Myeloid Leukemia

Chronic myeloid leukemia (CML) is a hematopoietic stem cell malignancy characterized by the presence of the (9; 22) (BCR-ABL1) fusion gene [104].

**Figure 3:**
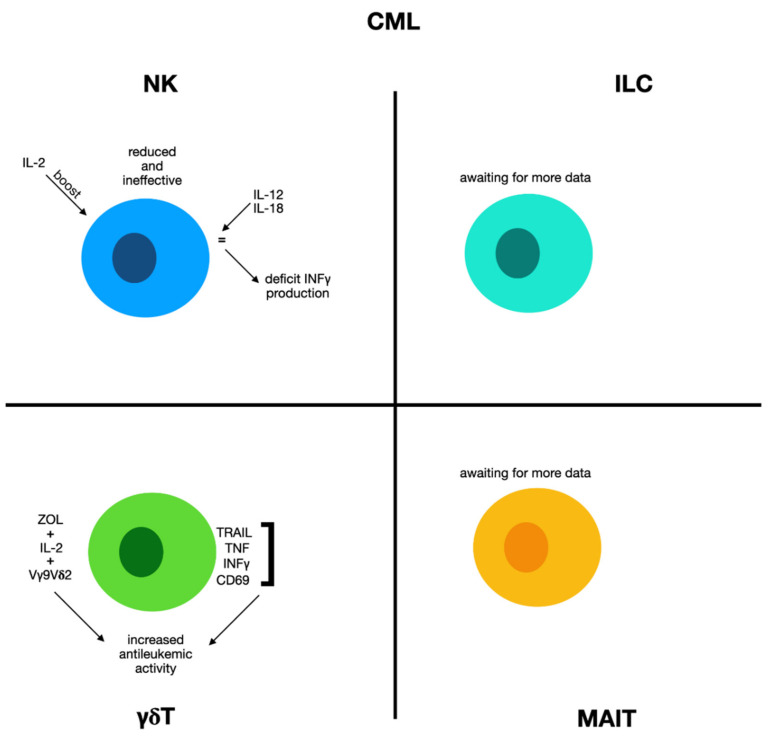
Chronic myeloid leukemia (CML) unconventional immune cell scenario. NK: NK cells are reduced and show alterations. IL-2 boost their activity. On the contrary, IL-12 and IL-18 diminish IFN-γ production. γδT displayed an increased presence of CD69, IFN-γ, TNF, and TNF-related apoptosis-inducing ligand (TRAIL), a stimulated phenotype, with an increased antileukemic activity. Once administered, Vg9Vd2 cells, ZOL, and IL-2 provoked a reduction in the leukemic burden.

In CML subjects, peripheral blood NK cells are reduced and show alteration in their growth and cytolytic ability with respect to normal donor blood NK cells [105]. In addition, the CD56+ bright NK subset and cytotoxic NK are remarkably decreased in all CML subjects. Nevertheless, this decreased toxicity was easily repristinated by incubation with recombinant IL-2. Authors demonstrated that NK clonogenic frequency and growth ability decrease as CML advances, revealing a hereditary defect in their ability to respond to physiological NK stimuli. Relevant modifications were reported in the absolute amount of circulating CD56+/CD3− NK and CD56+ bright NK, as well as growth on a per cell basis [105] (Figure 3).

NK cells can be active against a great group of hematological neoplasms comprising CML, and the rate and function of these immune effectors can be essential in the therapeutic response and prognosis of CML subjects [106,107]. In fact, an augmented percentage of mature NK cells have lately been reported to be correlated with successful imatinib withdrawal in the EURO-SKI trial. A portion of these subjects have been KIR-genotyped and catalogued according to haplotype B/x and A/A subtypes [108]. These data are validated by a diverse array of research, which evaluated the prognostic value of the KIR2DL5B genotype [109]. The receptor KIR2DL5 has a specific combination of genetic, and functional characteristics that give an inhibitory activity when joining to its ligand. The study was conducted on CML subjects inserted in two clinical protocols evaluating the possibility of TKI discontinuation: STIM and STIM2. Authors demonstrated that the KIR2DL5B-positive genotype was correlated to a late second molecular remission after TKI rechallenge, but not to time the first molecular remission [109]. These findings propose that KIR2DL5B could impact the lymphocyte-derived management of leukemic residual disease in subjects with CML relapse.

Analogous results were also reached by other experimentations that reported that KIR2DS1 was the only independent element for the minor possibility of attaining a complete cytogenetic response, minor progression-free survival, and overall survival [110], while Yeung et al. reported that KIR2DL5B was correlated with a minor event-free survival and with inferior major molecular response in a consecutive imatinib–nilotinib therapeutical approach [111].

#### 2.6.2. γδ Cells and Chronic Myeloid Leukemia

The acquisition of new knowledge in the field of the antineoplastic capabilities of γδ cells means that their employment in CML could be hypothesized [112]. When CML cells are cultured with zoledronic acid (ZOL) + IL-2 + IFN type I, their toxic effects are augmented and Vγ9Vδ2 cells may be able to effectively kill myeloid leukemia cells [113]. In this experimentation, γδ T cells displayed an increased presence of CD69, IFN-γ, TNF-α, and TNF-related apoptosis-inducing ligand (TRAIL), which demonstrates the achievement of a stimulated phenotype and increased antileukemic activity. Furthermore, stimulation with ZOL + imatinib has also been reported to augment the synapses with cytotoxic activity between Vγ9Vδ2 cells and leukemic cells in vitro [114]. To confirm these data, it was also shown that when Vγ9Vδ2 cells, ZOL, and IL-2 were administered in a leukemia animal experimental mouse model, they provoked a reduction in the leukemic burden in vivo and allowed a longer survival in these animals [114]. Thus, it is clear that the antitumoral activity of Vγ9Vδ2 T cells can be induced by specific drugs and molecules, even if the frequency and clinical significance during the progression of leukemia still remain controversial.

A diverse procedure to augment cell expansion in vitro was planned by generating Vγ9Vδ2 CD27/CD45RA double-negative effector memory cells [115]. Authors produced a Ph+ cell line, EM-2eGFPluc, and intravenous administrations of these cells into NOD. Cg-Prkdcscid Il2rgtm1Wjl/SzJ (NSG) animals caused a bone marrow engraftment. In vitro-expanded γδ T cells intraperitoneally survived at least 33 days post-injection. This administration caused a reduced bone marrow leukemia load in treated animals, and γδ T cells were identified in bone marrow and spleen, indicating the possible efficacy of this treatment [115].

### 2.7. Natural Killer Cells and ILCs in Chronic Myeloproliferative Diseases Ph−

**Figure 4:**
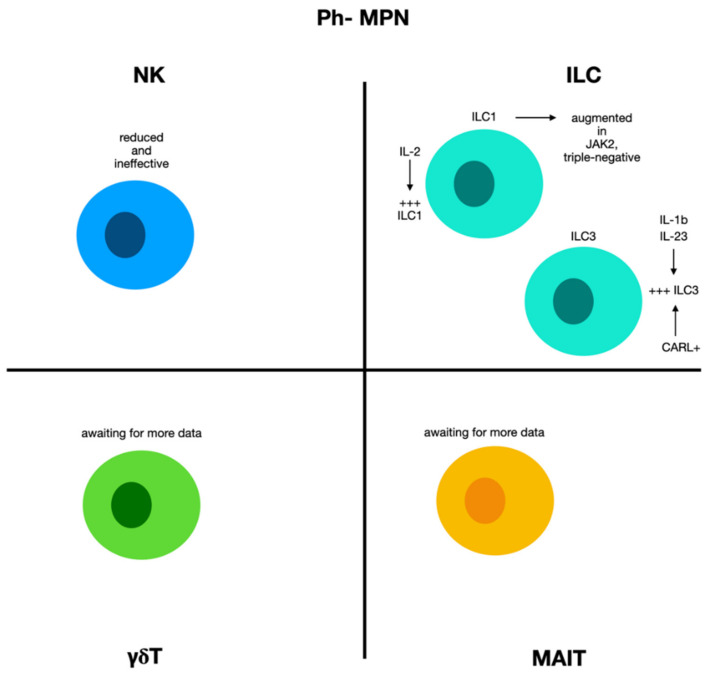
BCR-ABL-negative myeloproliferative neoplasms (Ph- MPN) unconventional immune cell scenario. NK: Ph- MPN patients have fewer NKs with altered function. ILC: ILC1 was augmented in JAK2-mutated and triple-negative patients, while ILC3 was increased in the CALR-mutated group. The ILC3 augment can be correlated with the increased concentration of IL-1b and IL-23.

BCR-ABL-negative myeloproliferative neoplasms (Ph− MPN) make up a various set of hematologic malignancies, comprising polycythaemia vera (PV), essential thrombocythemia (ET), and myelofibrosis (MF). Mutations in Janus kinase-2 (JAK2), calreticulin (CALR), and myeloproliferative leukemia protein (MPL) genes have been associated with these diseases [116] (Figure 4).

Ph- MPN patients have fewer NKs with altered function. Different studies performed on MF patients displayed a condition of mutation-dependent immune changes with cellular elements of the innate immunity exhibiting different types of functional inadequacy [117,118]. For instance, ILC1 was augmented in JAK2-mutated and triple-negative patients, while ILC3 was increased in the CALR-mutated group. However, regardless of the mutational condition, ILCs presented reduced activity. As for the mechanisms responsible of these alterations, the augment of ILC1 can be justified taking into consideration the augmented levels of IL-12 found in circulation. This interleukin is crucial for ILC1 maturation and ILC2 transformation into ILC1. Similarly, the ILC3 augment c be correlated with the increased concentration of IL-1b and IL-23. Of note, the number of ILC3 markers was considerably greater in Ph-MPN patients with an intermediate-2/high IPSS score, suggesting the possible effects in myelofibrosis progression [119].

### 2.8. Multiple Myeloma

#### 2.8.1. Natural Killer Cells and Multiple Myeloma

Multiple myeloma (MM) is a malignant disease of plasma cells that grow in protected niches in the bone marrow. While the outcome of MM improved in recent years because of novel treatment options, it remains an incurable malignancy [120,121].

**Figure 5:**
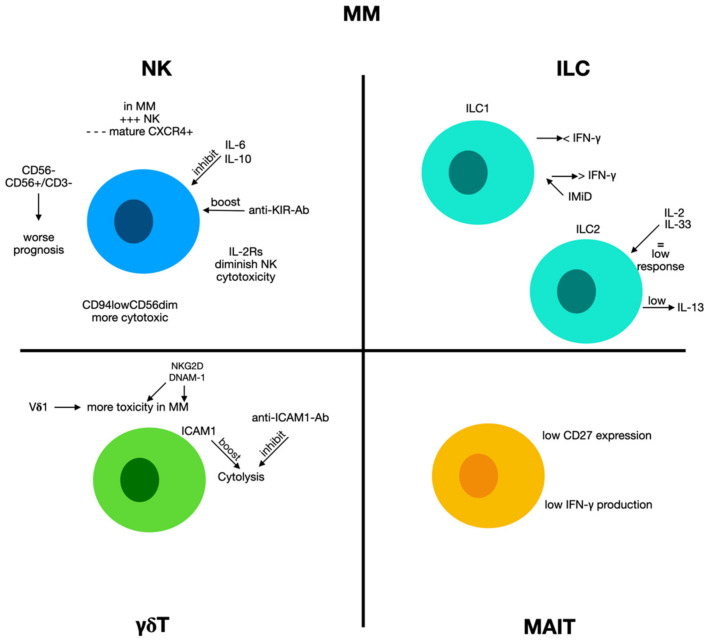
Multiple myeloma (MM) unconventional immune cell scenario. NK: MM subjects presenting a more significant number of NK cells at the onset of disease had a severer prognosis, leading to the unproductive stimulation of the immune system. Patients augmented with NK cells were concomitant with a less mature CXCR4+ NK cell subset. The presence of CD56+ CD3− NK cells of CD56- was related to a worse prognosis. IL-6 and IL-10 participate in NK cell functional alteration. Anti-KIR antibodies stimulated antineoplastic NK toxicity, as well as soluble IL-2 receptor. ILC: IFN-γ decreased in MM. The administration of IMiDs such as pomalidomide re-establishes the production of IFN-γ by ILC1s. ILC2s showed a weak ability to produce IL13 and an inadequate response to IL-2/IL-33 stimulation. γδT: IFN-γ secretion and Vδ1 T cell toxicity against MM cells occurred due to the T cell receptor (TCR) and other molecules (such as NKG2D, CD3, and CD2 receptors), DNAX accessory molecule-1, and intracellular cell adhesion molecule (ICAM)-1. MAIT presented low CD27 expression as well as low IFN-γ production.

Previous experimentations have verified an augmented amount of CD56+ CD3− NK cells in the bone marrow and peripheral blood in MM patients and subjects with monoclonal gammopathy of undetermined significance (MGUS), a clinical precursor condition leading to MM. Remarkably, MM subjects presenting more NK cells at the onset of disease had a more severe prognosis [122]. The augmented quantity of NK cells might be due to unproductive stimulation of the immune system containing MM cell proliferation [122,123] (Figure 5).

However, a recent meta-analysis showed that CD56-negative MM subjects showed a reduced OS in Asian subjects and reduced PFS in non-Asian subjects. It is noteworthy that not even the new therapeutic approaches are capable of modifying the unfavorable prognosis caused by CD56 negativity, except for MM subjects undergoing ASCT [124].

Numerous cytokines produced in MM, such as IL-6 and IL-10, participate in NK cell functional alteration. A previous study demonstrated that IL-6 can inhibit the cytotoxic activity of NK cells [125]. At the same time, IL-10 disturbs the generation of TNF- α and IFN-γ [126,127] and stimulates the occurrence of NK-resistant tumor phenotypes [128]. Furthermore, other different soluble components may reduce NK-derived antimyeloma abilities. The presence of COX-2 on MM cells stimulates the generation of prostaglandin E2 (PGE2) [126] which augments the concentrations of cyclic adenosine monophosphate (cAMP) and blocks stimulating impulses transduced by CD16, NCR, and NKG2D. This causes a reduction in NK cell cytotoxicity [125,129]. At the same time, the augmented concentrations of soluble IL-2 receptors, reported in the sera of MM patients, may also alter the stimulation of NK cells originating via IL-2 by T lymphocytes [130].

A study tried to assess the effects of NK cells on gammopathy progression. In many MGUS patients, authors reported an augment of NK cells concomitant with a less mature CXCR4+ NK cell subset, while subjects with reduced NK cell quantity in BM have low CXCR4 and high CX3CR1 levels [131]. As the two chemokine receptors have opposite effects on bone marrow (BM) NK cells, it is possible to hypothesize that chemokine receptor expression controls the NK cell BM distribution during gammopathy progression. In any case, in advanced phases of the disease, NK cells have been negatively related to MM progression, confirming that MM disturbs NK cell anti-tumor activity [132]. In active MM, NK cell function is further altered, and DNAM-1 and NKG2D expression is reduced, probably owed to the generation of soluble ligands that may stimulate receptor internalization, or to the effects of cytokines or exosomes produced by MM cells [133]. Thereby, inhibitory receptor expression, such as PD-1, is augmented and causes NK cell exhaustion after interaction with ligands, such as programmed cell death ligand-1 (PD-L1) expressed on MM cells [134].

However, as reported above, the relationship between NK cells and the progression of MM is debated, and disagreement is also reported regarding NK cell functions as either decreased NK cell functionality or augmented NK cell function. This is probably due to the clinical stage, and the different aggressiveness of the disease [135,136,137,138,139,140,141].

In a previous report, we evaluated NK characteristics in the bone marrow and peripheral blood of MM patients at the onset of the disease [142]. Our results demonstrated that NK cells were more frequent than in normal subjects. Among total MM-NK cells, we identified a relevant augment of the CD94lowCD56dim NK cell subset, which is already present in MGUS and smoldering MM, and ultimately expands during disease progression. Moreover, a relevant percentage of CD94lowCD56dim NK cells was in a growth condition. We evaluated these cells for their cytotoxic capabilities, including principal cytotoxic NK cells against autologous MM cells. In vitro, MM cells could quickly stimulate the growth of the CD94lowCD56dim NK cell subset. Mechanistically, this augment exists due to cell-to-cell contacts between MM and NK cells and necessitated both stimulations via DNAM-1 and interaction with CD56 present on MM cells [142].

Other studies have attempted to evaluate the processes of cytotoxicity in NK cells towards MM plasma cells. NK toxicity is related to the presence of activating receptors (AR) and inhibitory receptors (IRs) on the NK cell membranes connected with specific ligands present on target cells. Targeting NK IRs may inhibit NK cells from identifying and destroying myeloma cells. To overwhelm IR/AR disequilibrium and the modified stimulation after AR decrease, the employment of immune checkpoint inhibitors to block IRs on NK cells can diminish the inhibitory impulse, thus augmenting NK cell stimulation [143]. For this reason, numerous assays have tried to develop anti-KIR antibodies, and preclinical research demonstrated that the anti-KIR mAb 1-7F9 (IPH2101) reduced inhibitory receptors KIR2DL1/2/3 and stimulated antineoplastic NK toxicity against leukemic cells [144]. In MM, combined lenalidomide (Len) administration with IPH2101 in experimental animal models increased the effects of anti-MM NK cells and augmented MM cell clearance [145].

A phase I trial (NCT00552396) evaluated IPH2101 as a single drug in MM subjects and displayed augmented NK cell toxicity against MM cells ex vivo. The administration of IPH2101 seemed secure and unburdened with significant side effects at the dosage that attained complete inhibitory KIR saturation [146]. A different phase I trial (NCT01217203) performed by the same research group evaluated the IPH2101-Len combination, whereby numerous subjects had severe side effects, but some presented clinical responses [147].

#### 2.8.2. Innate Lymphoid Cells 1 and Multiple Myeloma

In patients with plasma cell dyscrasias, an augment in the fraction of ILC1s is reported in the bone marrow. Furthermore, though the ability of ILC1s to produce cytokines such as IFN-γ was conserved in MGUS subjects, it was remarkably decreased in smoldering MM patients [148]. Interestingly, ILC1 cells have been reported to produce augmented concentrations of Ikzf3 (Ajolos), a transcription factor implicated in B cell differentiation, which is a target of immunomodulatory (IMiDs) drugs. The administration of IMiDs such as pomalidomide re-establishes the production of IFN-γ by ILC1s [149], and the change in ILC1 function might be employed to treat multiple myeloma [150], increasing the effectiveness of drugs such as daratumumab, a monoclonal antibody directed against CD38.

In a different study, authors employed the KHYG1 NK cell line originating from a subject with an NK cell leukemia, i.e., CD38low, and can be modified to transitorily present a CD16 receptor variant coding the F158V polymorphism (CD16F158V). This condition increases daratumumab function by stimulating ADCC via the non-cleavable CD16 variant [151]. Equally, FcεRIγ-deficient NK (g-NK) cells are a rare group of cells originated from CMV-seropositive subjects (CD38low and SLAMF7low). An experimentation demonstrated that with respect to traditional NK cells, ex vivo g-NK cells, in combined administration with daratumumab, presented an augmented cytotoxicity against MM cells [152].

#### 2.8.3. Innate Lymphoid Cells 2 and Multiple Myeloma

In patients with monoclonal gammopathies, a reduced amount of ILC2s in the bone marrow was reported, and this finding was associated with a concurrent augment in the circulating set of ILC2s. In MGUS subjects, ILC2s showed the ability to produce IL13, which was not reported in subjects with smoldering MM [149]. Moreover, diverse experimentation demonstrated that myeloma cell proliferation was linked with modifications of activity and phenotypic changes in bone marrow ILC2s, with an augmented presence of maturation markers and decreased cytokine response to IL-2/IL-33 [153]. A study recognized a subset of KLRG1hi ILC2s stored in the spleen and liver of Il2rg/Rag2/mice reconstituted with BM ILC2s. An analogous set of KLRG1hi ILC2s was identified in the peripheral blood, spleen, and liver of IL-33-treated wild-type animals. The attendance of KLRG1hi ILC2s in ILC2-reconstituted Il2rg−/− Rag2−/− animals or in IL-33-treated wild-type animals, which were correlated with augmented eosinophil amounts but did not influence multiple myeloma progression. Remarkably, while reduced myeloma cell proliferation was displayed after IL-12 and IL-18 administration in Rag-deficient animals, this was overturned when animals were treated with IL-33 together with IL-12 and IL-18 [153]. These results suggest that IL-33 administration stimulates circulating inflammatory KLRG1hi ILC2s, blocks type 1 immunity against multiple myeloma cells, and contraindicates the therapeutic dispensation of IL-33 to myeloma subjects.

In the literature, there are no in-depth studies on the role played by ILC3 on the onset and progression of MM.

#### 2.8.4. γδ T Cells and Multiple Myeloma

Recent studies have proposed that γδ T cells may also be anti-myeloma effectors [154,155,156]. Stimulated γδ T cells exercised powerful toxic effects against MM cells in vitro [157,158], and represented a possible and secure strategy for MM treatment [159].

In an experimental study, Vδ1 T cells obtained from normal subjects showed pronounced toxicity against plasma cells derived from MM patient bone marrow [160]. Moreover, Vδ1 T cells derived from MM subjects displayed similarly relevant destruction of primary myeloma cells, as well as against myeloma cell lines U266 and RPMI8226 and plasma cell leukemia ARH77.

As far as the mechanisms are concerned, IFN-γ secretion and Vδ1 T cell toxicity against MM cells partially occurred via the T cell receptor (TCR) and other molecules (such as NKG2D, CD3, and CD2 receptors; DNAX accessory molecule-1; and intracellular cell adhesion molecule (ICAM)-1). Authors have stated that Vδ1 T cells are extremely myeloma-reactive and have proposed Vδ1 T cells as a possible agent for a new anti-myeloma immunotherapy [13].

Other researchers have verified these findings and attempted to further clarify the role of these cells. The stimulated T cells destroyed RPMI8226 and U266 myeloma cells in a γδ T cell dosage-dependent modality, and upon in vitro treatment with mevastatin or zoledronic acid, MM killing improved. Furthermore, of considerable interest is the fact that the amount of ICAM-1 on MM cells seems to correlate with the toxicity exerted by γδ T cells, and that the employees of an anti-ICAM-1 monoclonal antibody blocked cytolytic effects. From the point of view of a possible future therapeutic use, it is interesting to note that AMO-1 myeloma cells transfected with ICAM-1 cDNA, were vulnerable to γδ T cells, unlike parental AMO-1 cells. This indicates that MM subjects with cells presenting outstanding amounts of ICAM-1 are appropriate for cellular immune treatment employing γδ T cells in clinical settings [160]. The routine characterization of γδ T cells in samples of MM patients could work similarly to personalized medicine.

Recent attempts to join traditional treatments with immunotherapy for MM have led to the development of numerous encouraging therapies. Niu et al. reported that low-dose bortezomib did not reduce the survival of stimulated γδ T cells, but caused MM cell suicide [161]. Moreover, low-dose bortezomib augmented the presence of NKG2D and DNAM-1 ligands on MM cells, which increased the sensitivity of MM cells to the cytotoxic effects of γδ T cells. These findings indicate that the combined administration of low-dose bortezomib and stimulated γδ T cells encouraged synergistic killing activity on MM cells. This could also be the first of future studies to investigate this combination therapeutic approach. Many other drugs, such as carfilzomib, lenalidomide, and elotuzumab, augmented NK cell toxicity against MM cells [162,163,164,165], and the employment of stimulated γδ T cells along with chemotherapy drugs might be a promising strategy for the treatment of multiple myeloma.

#### 2.8.5. MAIT Cells and Multiple Myeloma

MAIT cells seem to be modified as quantities and functions in MM, although the data in the literature are sometimes discordant. A study stated that MAIT cell percentage in MM blood was decreased with respect to normal subjects, but it was equivalent to normal ageing subjects. Moreover, the authors did not find an augment of these cells in the bone marrow of these patients. MM patients at the onset of the disease displayed a decreased generation of IFN-γ and CD27 expression with MAIT cells, indicating an exhausted phenotype. However, IFN-γ production was re-established in relapsed subject samples.

Furthermore, the authors demonstrated that immunomodulatory drugs such as lenalidomide and pomalidomide could block MAIT cell activation [166]. These data were confirmed by other studies [167]. Thus, changes in MAIT cells can participate to the onset of disease and may be a novel immunotherapeutic objective for the therapy of MM.

### 2.9. Chronic Lymphocytic Leukemia

#### 2.9.1. Natural Killer Cells and Innate Lymphoid Cells in Chronic Lymphocytic Leukemia

**Figure 6:**
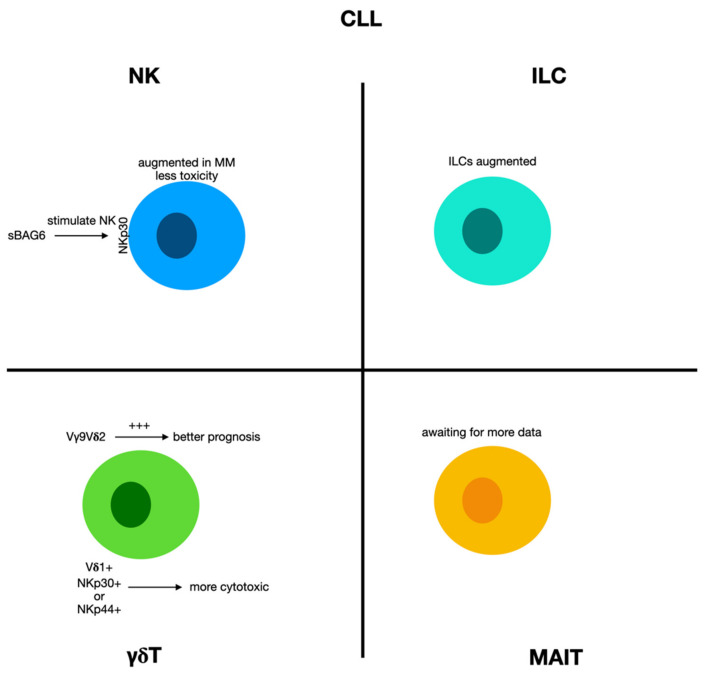
Chronic lymphocytic leukemia (CLL) unconventional immune cell scenario. NK: NKs are augmented but with less toxicity. Soluble BAG6 (i.e., NKp30-ligand) can stimulate NK cells via NKp30. ILC: ILCs are augmented. γδT: the increase in Vγ9Vδ2 tumor-infiltrating T cells was correlated with a favorable prognosis in CLL. Vδ1 cells that present NCRs could kill CLL cells via NKp30 and NKp44.

Immune disorders are characteristic of chronic lymphocytic leukemia (CLL), and, from an initial stage, immune alterations participate in infective or autoimmune complications [168,169]. Furthermore, the increasingly immunosuppressive milieu in CLL affects disease progression and treatment responses [170]. The processes triggering these conditions include both the innate and adaptive immune systems. In CLL patients, NK cells display reduced toxicity despite a quantitative augment [171]. NK cells are increased at the onset of CLL, and their amount seems to correlate with the outcome [172], although NK cells display an altered cytolytic function and a modified ability to produce cytokines [173,174] (Figure 6).

Among the probable processes accountable for this condition, it was reported that CLL cells may deliver soluble BAG6 (i.e., NKp30-ligand) and can challenge the exosome-bound BAG6 to stimulate NK cells via NKp30 [175].

Total ILC numbers were also remarkably augmented in the peripheral blood of CLL subjects with respect to age-matched normal subjects [176], and this boost showed a relationship with the leucocyte count in CLL subjects, proposing an increase in ILCs with CLL advancement as the number of ILCs showed an inverse correlation with time to first treatment. It is well known that the hypermutation status of immunoglobulin heavy-chain genes (IgVH) is one of the most relevant prognostic factors in chronic lymphocytic leukemia (CLL). According to the degree of IgVH mutation, CLL patients can be divided into two different prognostic groups. Nevertheless, despite the correlation with the outcome, the number of ILCs did not change between the two prognostically different groups, i.e., mutated (M-CLL) or unmutated (U-CLL) subjects, and the ILC subtype distribution of M-CLL subjects was equal to that of U-CLL [176].

#### 2.9.2. γδ T Cells in Chronic Lymphocytic Leukemia

γδ T cells have been stated to have augmented proportions in CLL subjects [177,178,179,180], although the techniques employed to ascertain the relative percentages of these cells have sometimes struggled to separate them appropriately from αβ T cells and NK cells.

A group of researchers perfected the computational CIBERSORT detection of tumor-infiltrating Vγ9Vδ2T cells by deconvoluting tumor microarray datasets, employing machine learning methods, and demonstrating more variability with respect to interindividual difference. Generally, the augment of Vγ9Vδ2 tumor-infiltrating T cells was correlated with a favorable prognosis in CLL [80].

In any case, the increased occurrence of these cells has been reported to be directly proportionate to leukemic advancement, as CLL subjects in more grave conditions displayed greater Vδ1 cell counts with respect to normal subjects. Thus, these lymphocytes are the principal T cell subset in the peripheral blood of these patients, and it was also reported that CLL patients presented Vδ1 cells with great concentrations of granzyme B [181]. These results propose that CLL can modify the γδ T cell occurrence and that these cells are effective during disease regression or advancement.

Furthermore, numerous aspects render γδ T cells as possible agents for novel treatments against CLL, given the great activity against leukemic cells and the lack of alloreactivity against the host.

Correia et al. reported that Vδ1 cells that present NCRs could kill CLL cells via NKp30 and NKp44. The use of low doses of IL-2 can appear to be helpful in maintaining NKp30 expression [182]. Furthermore, the expression of these NCRs was correlated with augmented concentrations of granzyme B and appear to have synergistic effects, causing higher cytotoxicity against CLL cells. So, some studies tried to identify elements to enhance the activity of these cells. Lança et al. showed that the expression of UL-16-binding protein 1 (ULBP1,) a ligand that activates the receptor NKG2D in CLL cells, is augmented in leukemic patients and is essential for the identification of Vδ1 cells [183], contributing to the antileukemic immune response.

Other studies have sought to elucidate the role of γδ T cells after drug administration for CLL treatment. One study used chemotherapeutic drugs and kinase inhibitors to augment the cell sensitivity to γδ T cell cytotoxic activity in CLL cells [184]. Numerous experimental models suggested synergistic actions of the combined administration of chemotherapy and adoptive transfer allogeneic Vδ2 T cells [185]. For instance, ibrutinib has been recognized to stimulate γδ T cells against CLL cells, and it was demonstrated as being capable of stimulating an antitumor phenotype [184]. It is known that γδ T cells from CLL are dysfunctional in the generation and activity of cytokines [186]. However, when Vγ9Vδ2 cells were treated with ibrutinib and IL2, a Th1 phenotype and memory cells were stimulated, and anti-leukemic activities were re-established [184].

### 2.10. Unconventional T Cells in Lymphomas

Innate immune responses are essential in the progression of Hodgkin lymphoma (HL) as Hodgkin Reed–Sternberg cells can elude immune-mediated identification and elimination with the augmented expression of PD-L1. It is known that patients with HL present an alteration of immune surveillance mechanisms. This condition could be partly responsible for the onset of the disease and its progression. As evidence of this, an increase in γδ T cells was found in these subjects. In the same subjects, there was an increase in the soluble component of MIC-A (s-MICA). Such data could also have prognostic significance, as NKT cells higher than 40 μL and high values of s-MICA appear to correlate with a worse prognosis.

Diffuse large B cell lymphoma (DLBCL) is the most common type of aggressive non-Hodgkin lymphoma. Based on the gene profile, it is possible to distinguish two different forms of the disease: one that originates from the germinative center (GC) and those originating from activated B cells (ABC). The two forms have a different prognosis and respond differently to standard therapy. The different behavior could at least be attributable to the different arrangement of the cells. Although in both forms, the Vδ1 T cells were the principal γδ T cell subset of cells and the GC type was associated with an increase in Vδ1+ T cells in tumors, whereas the non-GC subtype was related to a minor amount of γδ T cells [187].

Furthermore, while circulating Vδ1+ T cells of patients displaying a naïve phenotype, the more significant part of tumor Vδ1+ T cells exhibited a central memory phenotype. However, circulating resident γδ T cells from DLBCL subjects were not altered as far as their functionally is concerned, as they could produce great quantities of IFN-γ.

This observation suggests that the tumor environment in DLBCL patients is less favorable for the differentiation of Vδ1+ T and Vδ2+ T cells into the T effector memory cells subset, which could potentially exert a more potent antitumor response [188].

The existence of a correlation between immune cells and prognosis has been confirmed by other studies, supporting the idea that the immunological composition of the tumor microenvironment is capable of influencing a different evolution of the lymphomatous disease [189].

## 3. Therapeutical Perspectives and Concluding Remarks

It is now evident that numerous cells that participate in the regulation of innate immunity, especially NK T cells, have a crucial impact on anti-neoplastic protection due to their ability to drive the adaptive immunity towards a Th1 response which is advantageous for the control of malignancies. However, they may also negatively affect neoplastic advancement, especially after changes caused by the tumor milieu.

Recently, several strategies for modulating and augmenting the efficacy of the effectors of innate immunity have been suggested as a possible treatment for hematological malignancies.

A promising modality to augment NK cells infusion therapeutic potential is provided by producing bi- and tri-specific killer engagers (BiKEs and TriKEs). These molecules enclose variable portions from antibodies which can recognize diverse antigens. Indeed, these new therapies target antibody-dependent cell-mediated cytotoxicity (ADCC) mechanism by improving its efficacy on human NK cells. Indeed, ADCC is mediated by CD16 (FcγRIII), the low-affinity receptor for IgG Fc expressed in NK cells and other innate cells, leading to cytokine production and a cytotoxic response through the encounter of the Fc portion antibodies. One of these molecules was expected to identify antigens, such as CD19 or CD33, and was able to augment the degranulation of NK cells and the generation of cytokines against B-ALL and B-CLL or MDS primary cells [190]. In the following period, more complex constructs were designed. For instance, an IL-15 cross-linker TriKEs was built to recognize the antigen CD33 that augmented NK cell toxicity against the acute promyelocytic leukemia cell line HL-60, and against AML blasts. Furthermore, this technique helped to boost NK cell growth and survival, as well as increase the anti-leukemic activity in vivo [191]. This product is under evaluation in phase I clinical trials (NCT03214666). Following the same research path, a TriKes against the antigen CD19 was designed and tested against primary CLL cells in vitro [192]. The same research group also designed a new CLEC12A TriKE, i.e., a compound which can target an antigen on leukemic stem cells and AML cells. This construct displayed promising anti-leukemic in vivo efficacy in an animal xenograft model of HL-60 cells or primary AML cells cocultured with NK cells from healthy subjects [193]. The effectiveness of these killer engagers in augmenting NK cell anti-leukemic activity could guarantee the development of an effective treatment for a pathology that has not yet found resolutive treatments.

Several other attempts have been made to enhance the antitumor activity of the effectors of innate immunity in unconventional T cells. For instance, a high dose i.v. of vitamin C may also be evaluated to augment the in vitro proliferation and effectiveness of γδ T cell therapy [194,195].

Furthermore, the discovery that T and NK cells can present PD1 made it possible to hypothesize a new type of intervention in the treatment of HLA-I-defective malignancies, which are imperceptible by T cells, by employing monoclonal antibodies that target PD1 or PD-L1. Furthermore, NK cells can be controlled by inhibiting KIR or NKG2A, thereby overcoming inhibitory activity after their relationships with leukemic cells presenting HLA-I [196]. In the haplo-HSCT condition, it makes sense to alloreactive all donor NK cells. Several authors have investigated adoptive NK cells transferal to enhance anti-leukemic immune response after HSCT. Allogenic NK cells from normal subjects have the great advantage of being grown up in a non-immunosuppressive milieu, and their transference to leukemic subjects might represent a new interesting therapeutic approach [197]. Furthermore, donor NK cells have mismatched inhibitory receptors for HLA class I ligands, which permits them to target leukemic cells presenting ligands for their receptors, but not normal tissues, thus allowing the graft-versus-leukemia effect to avoid GVHD [56]. In vivo, a dose–response relationship was observed between the amount of NK cells administered in AML, CML, and MDS patients and a slower disease advancement or augmented relapse-free survival [198].

Other clinical trials have employed γδ T cells to destroy different leukemic cells, and one possible strategy to augment γδ T cell-mediated cytolysis is via the augmentation of NKG2D ligands on leukemic cells, which can be attained via diverse systems, such as proteasome inhibition and epigenetic change. In a study, NKG2D ligand expression was evaluated in Nomo-1 and Kasumi-1, two AML cell lines, after treatment with different amounts of bortezomib. Proteasome inhibitors remarkably augmented the expression of the NKG2D ligand ULBP [199]. The combined employment of γδ T cells and bortezomib caused higher cytotoxicity against AML cell lines than γδ T cells alone, and the same results were attained after employing T-ALL cells. Bortezomib augmented ULBP expression in T-ALL cell lines and enhanced the cytotoxic activity of γδ T cells against leukemic cells. So, it is possible to conjecture that employing a combined administration of a stress ligand-inducing drug with allogeneic γδ T cells originating from a normal donor can efficaciously destroy different neoplastic cells in hematological malignancies.

The variation of innate immunity can also be helpful in the field of vaccination treatment. Hematologic malignancies commonly present the required apparatus for inducing an antineoplastic immune response; nevertheless, several hematologic tumors are weakly immunogenic [200,201,202].

Mattarollo et al. tried to enhance the immune response against B cell lymphomas by generating a tumor cell vaccine, including galactosylceramide (-GalCer), that modifies the immune-adjuvant abilities of NKT cells [203]. In an experimental animal model employing E- myc transgenic mice, a single vaccination of irradiated -GalCer-loaded autologous lymphoma cells was adequate to block the proliferation of tumors and augment animal survival. Vaccine administration caused increased the IFN-γ production and transitory proliferation of NKT cells, the more relevant font of IFN-γ.

However, this therapeutical strategy was attempted in numerous other hematologic malignancies and was efficacious against AMLETO9a acute myeloid L=leukemia. In this case, -GalCer was substituted with mannosylceramide, causing long-lasting defense against tumoral cells [203]. These findings confirm the decisive immune-adjuvant activity of NKT cell ligands in therapeutic antitumoral vaccination against oncogene-driven lymphomas.

In the near future, a promising field of study could be used to employ immune cell-originated extracellular vesicles (EVs), which have some specific characteristics, e.g., comprising stability, the ability to cross blood–tumor barriers, and the ability to insert several effector molecules. For these reasons, immune cell-originated EVs are essential facilitators of intercellular communication that control numerous innate immune response systems and have been employed as potent antileukemic vaccines or for the treatment of hematologic malignancies [204].

However, despite the encouraging results obtained, the road to routine clinical use of the modulation of innate immunity in the treatment of hematological neoplasms still appears long, and numerous data are still needed to obtain a complete picture of the activity of these cells in hematological neoplasms. Further studies should aim to clarify some contradictions between the results obtained from different studies in the literature. In some cases, these differences may be due to the studies’ different experimental conditions, and the different influence exerted by the neoplastic cells on the immune system response. In other cases, methodological errors and inadequate sampling may lead to unreliable results. Furthermore, more experimentations should be dedicated to elaborating innovative in vitro models, such as three-dimensional spheroid cultures, which permit more reliable results with respect to traditional two-dimensional tumor γδ T cell cocultures [205].

Still, there are several difficulties in ascertaining the best therapeutic situations that take advantage of the full antineoplastic ability of these cells in vivo.

In a short time, investigations should aim to explore the clinical employment of combined approaches that might act on the neoplastic cells and the tumor milieu. Ultimately, the findings of these studies should provide essential comprehensions that can hopefully lead to novel therapeutic options to avoid relapse and enhance survival in hematologic patients.

## Data Availability

Not applicable.
